# Release and Demand of Public Health Information in Social Media During the Outbreak of COVID-19 in China

**DOI:** 10.3389/fpubh.2021.829589

**Published:** 2022-02-10

**Authors:** Songjia Tang, Xiaoxin Wu, Jingjing Chen, Fangfang Lu, Zhihao Zhang, Yingying Xu, Jufang Zhang

**Affiliations:** ^1^Plastic and Aesthetic Surgery Department, Affiliated Hangzhou First People's Hospital, Zhejiang University School of Medicine, Hangzhou, China; ^2^State Key Laboratory for Diagnosis and Treatment of Infectious Diseases, The First Affiliated Hospital, Zhejiang University School of Medicine, Hangzhou, China; ^3^Zhijiang College of Zhejiang University of Technology, Shaoxing, China; ^4^School of Computer Science and Technology, Shanghai University of Electric Power, Shanghai, China; ^5^School of Economics and Management, University of Science and Technology Beijing, Beijing, China

**Keywords:** information supply, information demand, economy recovery, text mining, social media, COVID-19

## Abstract

Information release is a key to the macro-economy during the outbreak of the Coronavirus Diosease-2019 (COVID-19). To explore the relationship between information supply by the government and public information demand in the pandemic, this study collected over 4,000 posts published on the most popular social media platform, i.e., WeChat. Many approaches, such as text mining, are employed to explore the information at different stages during the pandemic. According to the results, the government attached great importance to the information related to the pandemic. The main topics of information released by the government included the latest situation of the pandemic, announcements by the State Council, and prevention policies for COVID-19. Information mismatch between the public and Chinese governments contributed to the economic depression caused by the pandemic. Specifically, the topics of “the latest situation” and “popular scientific knowledge regarding the pandemic” have gained the most attention of the public. The information demand of the public has changed from the pandemic itself to the recovery of social life and industrial activities after the authority announced the control of the pandemic. However, during the recession phase, the information demand has shifted to asymptomatic infections and global pandemic trends. By contrast, some of the main topics provided by the government, such as “How beautiful you are,” were excessive because the public demand is insufficient. Therefore, severe mismatches existed between information release of the government and public information demand during the pandemic, which impeded the recovery of the economy. The results in this study provide strategical suggestions of information release and opinion guidance for the authorities.

## Introduction

Public emergencies are of extreme destruction and highly uncertainty, which could seriously endanger people's health, safety, and social activities. The outbreak of Coronavirus Disease-2019 (COVID-19) has caused more than 26 million infections and 5,000,000 deaths as of December 1, 2021 reported to WHO (1). Currently, it continues to spread around the world. Obviously, it has become the largest challenge since World War II and is causing immeasurable influence on the economy and society. Public emergencies are often accompanied by the spreading of false information (2–4). With the engagement of social media tools, rumors regarding the pandemic could spread earlier, faster, and wider than that in a traditional manner. It could cause public panic easily, which raises challenges for the governments to carry out pandemic prevention and crisis management (5, 6). Therefore, timely and accurate information release is crucial to maintain social stability during the outbreak of COVID-19 (7–11).

The release of official information during the COVID-19 outbreak has been investigated. Some researchers paid attention to the information release of the official government website and made a systematic evaluation on specific data. For example, Weng et al. facilitated the data-mining technology to analyze the information transparency of provincial and municipal governments. A systematic quantitative evaluation of the quality on the data openness of government was conducted in (12). Zhang et al. summarized the highlights of information release of the COVID-19 pandemic and emphasized the deficiencies in the process of information releasing (13). Zhong selected a series of press conferences held in five key provinces (cities) of epidemic prevention and control in Hubei, Beijing, Shanghai, Guangdong, and Zhejiang in the first half of 2020 as the research object, analyzed the dialog practice performance of local governments in response to sudden public crises, and pointed out the shortcomings of poor dialog opportunity, inadequate dialog ability, and insufficient verbal interaction in local governments (14).

Some researchers also paid attention to the information released on the official WeChat run by governments. For example, Peng et al. took “Zhuhai Release” as an example to investigate the information release of the pandemic and its impact, and Peng pointed out that these government-run WeChat official accounts should play a leading role in public health emergencies and adhere to the concept of audience-oriented release (15).

However, the current research on the information release of government-run WeChat official account is still in the infancy phase. The data mining on the text of the government-run WeChat official account, with great advantages in exploring the interactive patterns between the government and the public, could be used to conduct quantitative research on the release of government information and to depict the public reading patterns and preferences. Combining with the posts published by the government-run WeChat official account “Health China,” we analyzed the information supply and the characteristics of the public information demand using text mining and clustering methods from the perspective of information supply and demand.

## Materials and Methods

### Data Source

During the period of public health emergencies, the timely and accurate information released from the National Health Commission plays an extremely important role in reporting the dynamic situation of the pandemic and preventing the spreading of the pandemic. Compared with other government departments, the public will pay more attention to the information released by the National Health Commission. Moreover, WeChat is the dominant social media tool for the public in China to obtain public information. However, the various sources of information in WeChat usually make it difficult for the public to distinguish the authenticity, which could not meet the urgent needs of the public for effective information during the pandemic. Moreover, the spreading of rumors could easily lead to a secondary disaster of the information pandemic. In contrast, as the official information release account of the government, the information released by government WeChat is considered to be authoritative and reliable. In the complex environment of “Personal Media,” a government-run WeChat official account, as a source of information, is easier to obtain the trust of the public, as it represents the official voice and position of the government.

The National Health Commission is the administrative department of pandemic prevention and control. In this context, we followed the WeChat official account “Health China” by National Health Commission and collected all the data of the account from January 01, 2020 to March 31, 2020 for analysis.

### Methods

In journalism and communication, the demand for social news is an important factor that affects the amount of information in news. Similarly, in this paper, to measure the supply-demand ratio of articles, we used normalization to dimensionless the number of published and “like” of the article. The normalization formula is as follow:

(1) $$\begin{array}{r} X_{\text{norm }}=\frac{X-X_{\text{min}}}{X_{\text{max}}-X_{\text{min}}} \end{array}$$

**Figure 1:**
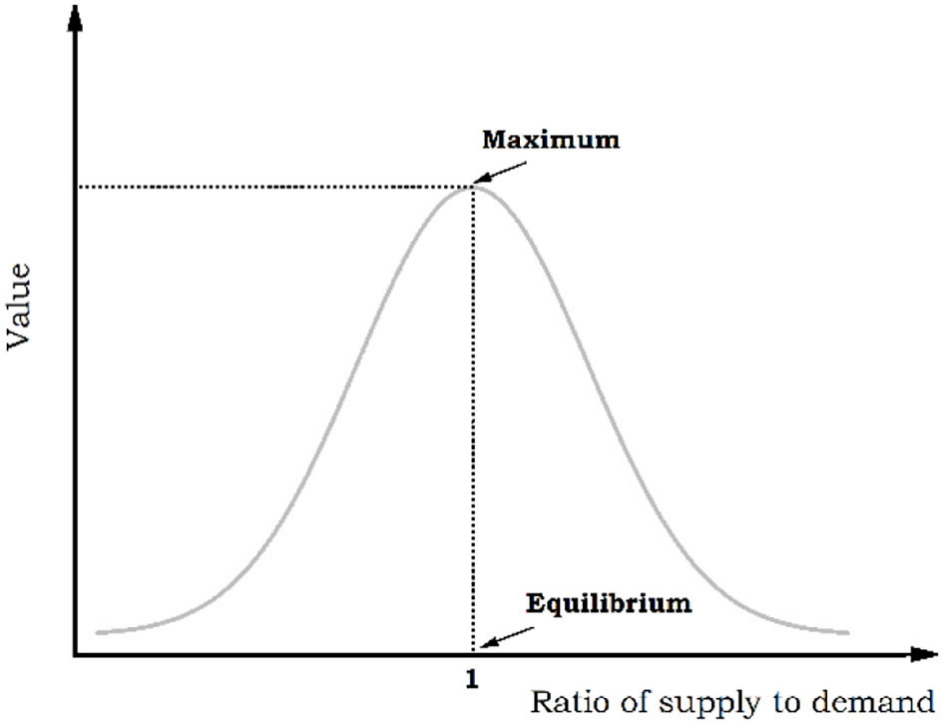
The curve describing the relationship between news value and supply-demand ratio.

Where *X*_norm_ is the normalized data, *X* is the original data, with *X*_*max*_ and *X*_*min*_ denoting the maximum and minimum value of the original data, respectively. The ratio of supply to demand of posts is used as a critical factor influencing their value, and the relationship between them is shown in Figure 1.

When the supply of information, i.e., posts in this context, is less than the demand, it means that released information cannot meet the demand from the public, therefore, the overall value is at a low level. When the supply of posts exceeds the demand, it indicates that the public is not interested in the content of the posts or the information contained in it is of low importance, so the value of posts is relatively low. These posts can only be of maximum value if supply and demand are balanced. Based on VOSviewer bibliometric analysis software, we extract the subject words in the posts of government-run WeChat official account “Health China,” then counts the occurrence of them, making cluster analysis to present the distribution hot spot map of the subject words in each stage. At the same time, the number of posts containing the subject word with high frequency is chosen as supply, the average number of posts “like” is chosen as demand to further analyze the relationship between supply and demand.

## Findings

From January 01, 2020 to March 31, 2020, the total number of posts by WeChat account “Health China” is 2,307. As shown in Table 1, these posts published during such a period are grouped by the evolution processes of the pandemic (i.e., incubation period, concentrated outbreak period, stable control period, and recession period), which are subject to the development of the pandemic and key events.

**Table 1:** The milestones.

**Pandemic stage**	**Date range**	**Date**	**Key event**
Initial	01/01–01/19	01/20	Prof. Zhong affirmed the fact of human-to-human transmission of COVID-19
Outbreak	01/20–02/15	02/15	Official Press announces: Be positive
Under control	02/16–03/05	02/16	Announcement for the resumption of work and production
Recession	03/06–03/31	03/06	Announcement of the policies for preventing the imported foreign cases

**Figure 2:**
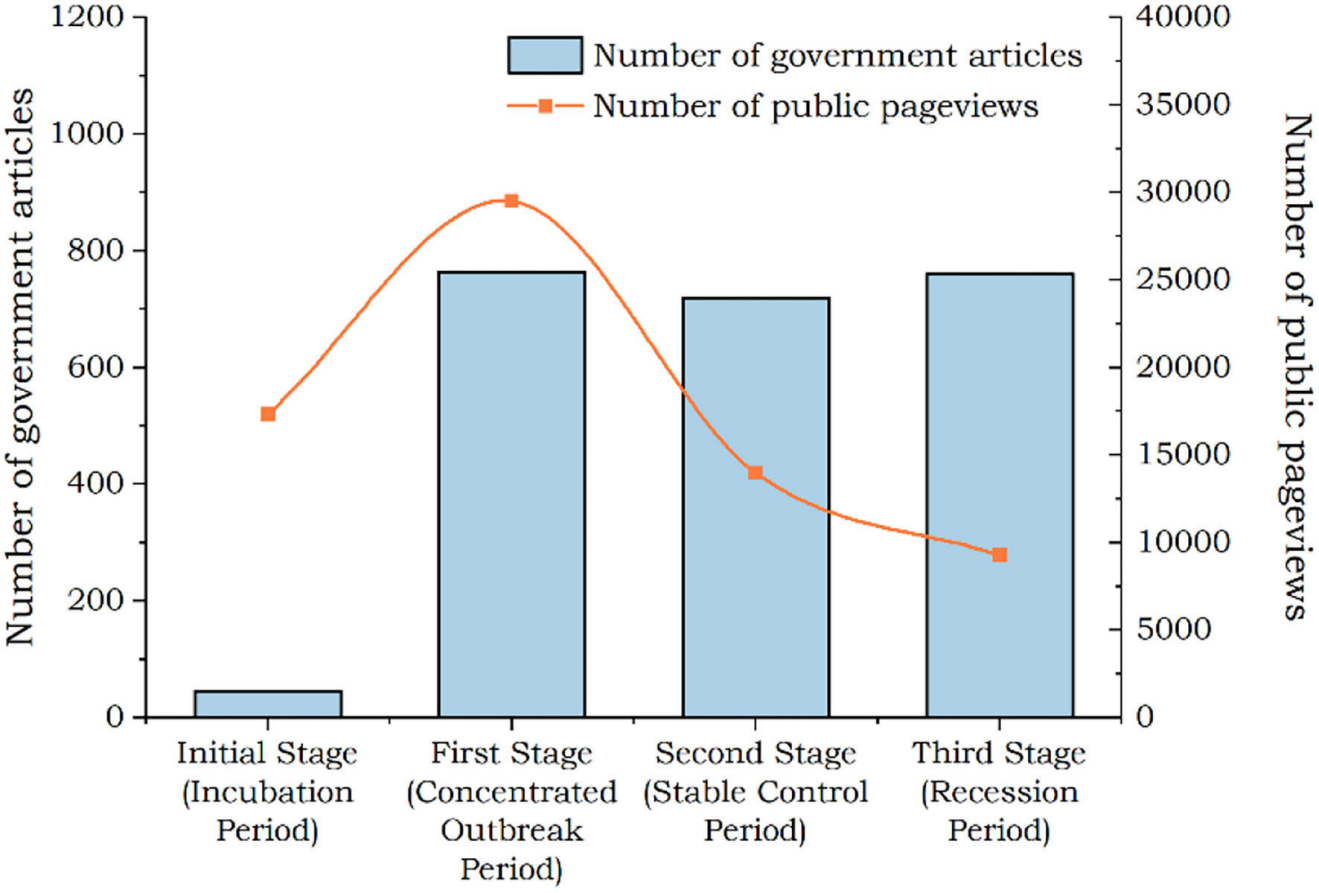
Cluster visualization of key words in government WeChat posts in four stages of epidemic situation.

**Figure 3:**
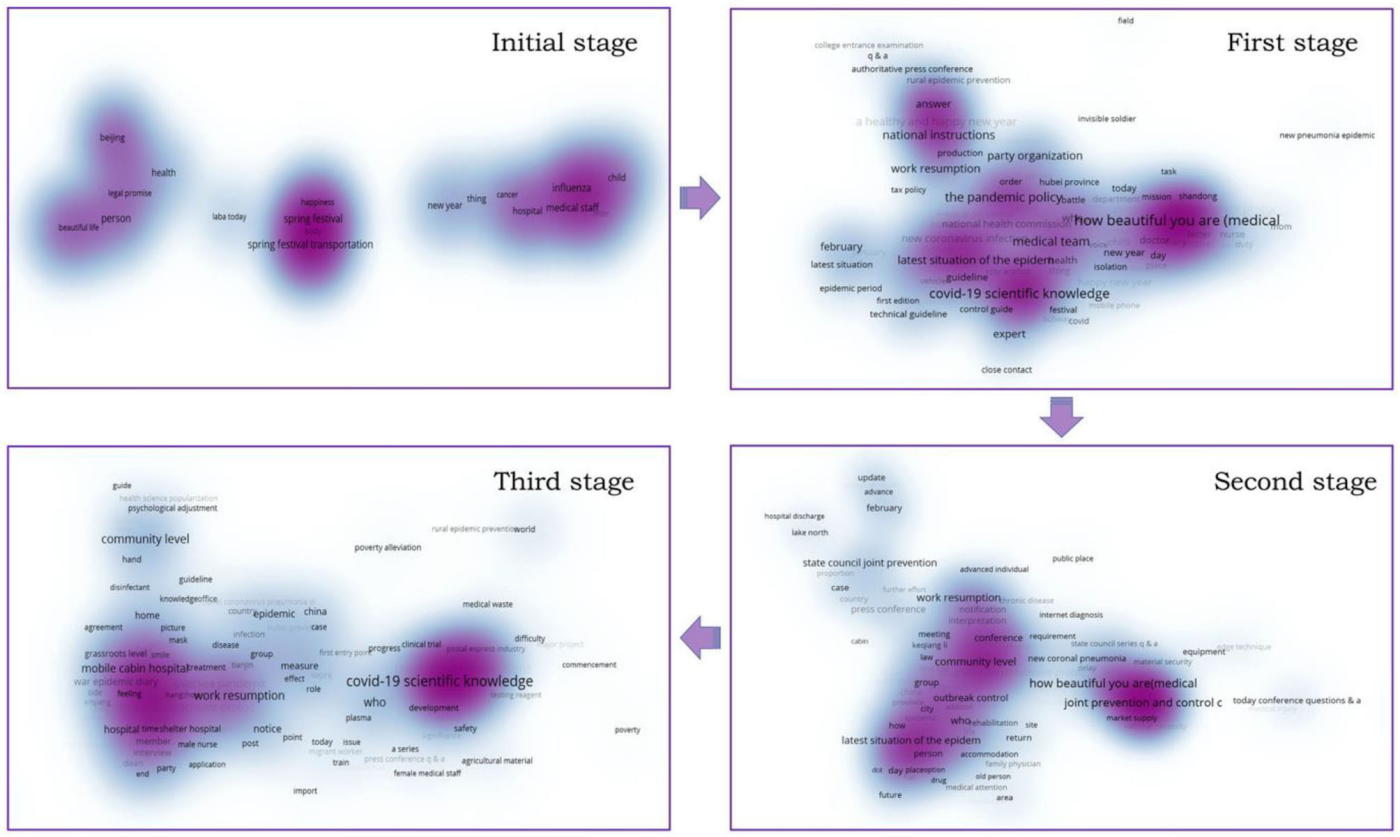
The trend of no. of posts and no. of page views by the public in each stage.

The government's supply of information and the public's information demand had significantly changed at different stages. We performed a detailed analysis according to text data. In terms of information supply, the number of posts had increased sharply from the incubation period to the outbreak period. The number of posts had decreased slightly from the outbreak period to the control period. The number had rebounded slightly from the control period to the recession period (as shown in Figure 2). At the same time, we clustered the keywords of government tweets at each stage. As shown in Figure 3, in the outbreak stage, the subjects of government information release are relatively concentrated, while in other stages, they are relatively scattered. In addition, we found that after the outbreak of the epidemic, the information released by the government had closely revolved around the epidemic.

### Supply and Demand of Public Health Information During Progression Period

#### Information Supply From the Government

During the progression period of the pandemic (from January 01, 2020 to January 19, 2020), the number of government posts is 46 and 3 posts are related to COVID-19, accounting for only 6% of all the posts. These metrics describing the posts are presented in Table 2.

**Table 2:** WeChat posts regarding COVID-19.

**Published date**	**Title**	**No. of views**	**No. of “like”**
01/11	China will share information about the novel coronavirus gene sequence with the World Health Organization	10,739	106
01/15	Expert groups from Hong Kong, Macao, and Taiwan visited Wuhan	9,164	31
01/19	The National Health Commission has actively carried out the prevention and control of novel coronavirus infection	68,974	452

The National Health Commission released the first post of COVID-19 on January 11, 2020: “China will Share Novel Coronavirus Gene Sequence Information with the World Health Organization,” with 10,739 pageviews. The second post, “Expert groups from Hong Kong, Macao, and Taiwan visited Wuhan,” released on January 19, was viewed 9,164 times. The third post was released on January 19, “The National Health Commission has actively carried out the prevention and control of novel coronavirus infection,” and obtained 68,974 views, which highly attracted the public's attention.

During the progression period, the posts from “Health China” included multiple themes, such as influenza, spring festival, hospital, medical staff, transportation in spring festival, and Beijing.

#### Public Information Demand

In the progression period, the public attention was more diverse according to the top 5 most viewed posts by “Health China” which are presented in Table 3. The main keywords were: health conference, medical dispute, scientific knowledge popularization, medical staff, health city, influenza, and cancer.

**Table 3:** Posts attracting the attentions of the public (top 5).

**Public focus**	**Representative posts**	**Release date**	**Content**
High-level meetings	The 2020 National Health Work Conference was held in Beijing	01/07	Work in 2019 was summarized, the construction of the health system was studied and strengthened, key tasks in 2020 was planned.
High-level deployment	The National Health Commission has actively carried out the prevention and control of novel coronavirus infection	01/19	The Health Commission had set up a leading group for pandemic to guide and support Hubei Province and Wuhan and to carry out treatment, pandemic prevention and control, and emergency response.
Medical dispute incident	Sun Wenbin was sentenced to death	01/16	Sun Wenbin intentional homicide case (medical dispute incident), the defendant Sun Wenbin was sentenced to death for intentional homicide.
Medical staff	In order popularize scientific knowledge, medical staff is talented!	01/09	Guangdong Dongguan medical version of “Wild Wolf Disco and other songs were released to popularize medical scientific knowledge.”
Medical staff	Attention to the medical students! The registration of 2020 National Physician Qualification Examination will start tomorrow	01/08	Registration of the physician qualification examination will start nationwide in 2020.

According to Table 3, in the progression period, the government supply of information about COVID-19 is limited, and the public attention is also at a low level. Therefore, both the government and the public were in a state of neglect, which laid hidden dangers for the outbreak and rapid spreading of the pandemic.

### Supply and Demand of Public Health Information During the Outbreak Period

During the outbreak period from January 20, 2020 to February 15, 2020, the number of new confirmed cases were increased rapidly. China carried out a key measurement to stop the spreading of the virus and resolutely closed the channel leaving from Wuhan.

The number of WeChat posts by “Health China” was 762, and the average number of daily posts was 30, which was 12 times the daily number during latency. There were 740 posts involving COVID-19, making up 97% of all the posts. It is obvious that the Chinese government attached great importance to COVID-19 during the outbreak period. Meanwhile, there were 72 posts that achieved over 100,000 views, 421 posts with 10,000–100,000 views, and posts with more than 10,000 views accounted for about 66% of the pandemic-related posts. Table 4 shows the featured posts of top-5 hot themes during the outbreak period. It is observed that the public paid great attention to the information related to the pandemic.

**Table 4:** Featured posts of top-5 hot themes during the outbreak period.

**Public focus**	**Representative posts**	**Release date**	**Primary content**
Pandemic prevention policies	Announcement of the National Health Commission of the People's Republic of China	01/21/2020	Novel coronavirus pneumonia would be included in the management of Class B infectious diseases and quarantinable infectious diseases, prevention and control measures were taken the same as Class A infectious diseases.
Scientific knowledge propagation	Authoritative books on pandemic prevention are coming! Read this guide carefully	01/30/2020	The Public Guide for the Protection of Novel Coronavirus Disease compiled by the Chinese Center for Disease Control was published
Diagnosis and treatment protocol	Notice on the Issuance of the Diagnosis and Treatment Plan for COVID-19 (Trial Fourth Edition)	01/28/2020	The phased diagnosis and treatment protocol of COVID-19
Medical staff	[How beautiful you are] A song for the white warriors who try their best to prevent and control the pandemic	01/25/2020	The song, “How Beautiful You Are” was jointly produced by the National Health Commission and the Chinese Medical Doctor Association. Expressing admiration for medical workers and recorded on New Year's Eve.
Pandemic prevention policies	Notice on Strengthening the Prevention and Control of Novel Coronavirus Pneumonia Pandemic in Communities	01/25/2020	Pandemic prevention and control strategies and specific measures were put forward at three levels: no cases are found in the community, cases or outbreak in the community, and pandemic transmission in the community

There were 10 core keywords extracted from information supplied by the government (as presented in Table 5). The top five keywords were: “how beautiful you are (medical staff),” “COVID-19 scientific knowledge,” “joint prevention and control conference,” “the pandemic policy and national instructions.”

**Table 5:** The frequency of government release of outbreak posts and the frequency of public reading preferences.

**Number**	**Keyword**	**Government**	**Public**
1	How beautiful you are (medical staff)	227	6
2	COVID-19 scientific knowledge	168	41
3	Joint prevention and control conference	144	3
4	The pandemic policy	74	31
5	National instructions	55	6
6	A healthy and happy New Year	37	5
7	Medical team	35	0
8	Anti-pandemic	23	0
9	Latest situation of the epidemic	22	22
10	Party organization	14	0

Compared with the diversified themes of government information supply, the public had only paid high attention to certain themes. During the outbreak period, there were 152 posts with high public attention. The top three themes that gained most of the public's attention were COVID-19 scientific knowledge, pandemic policies, and the latest situation of the pandemic. The top five posts that gained most peoples' attention during the outbreak period were listed.

**Figure 4:**
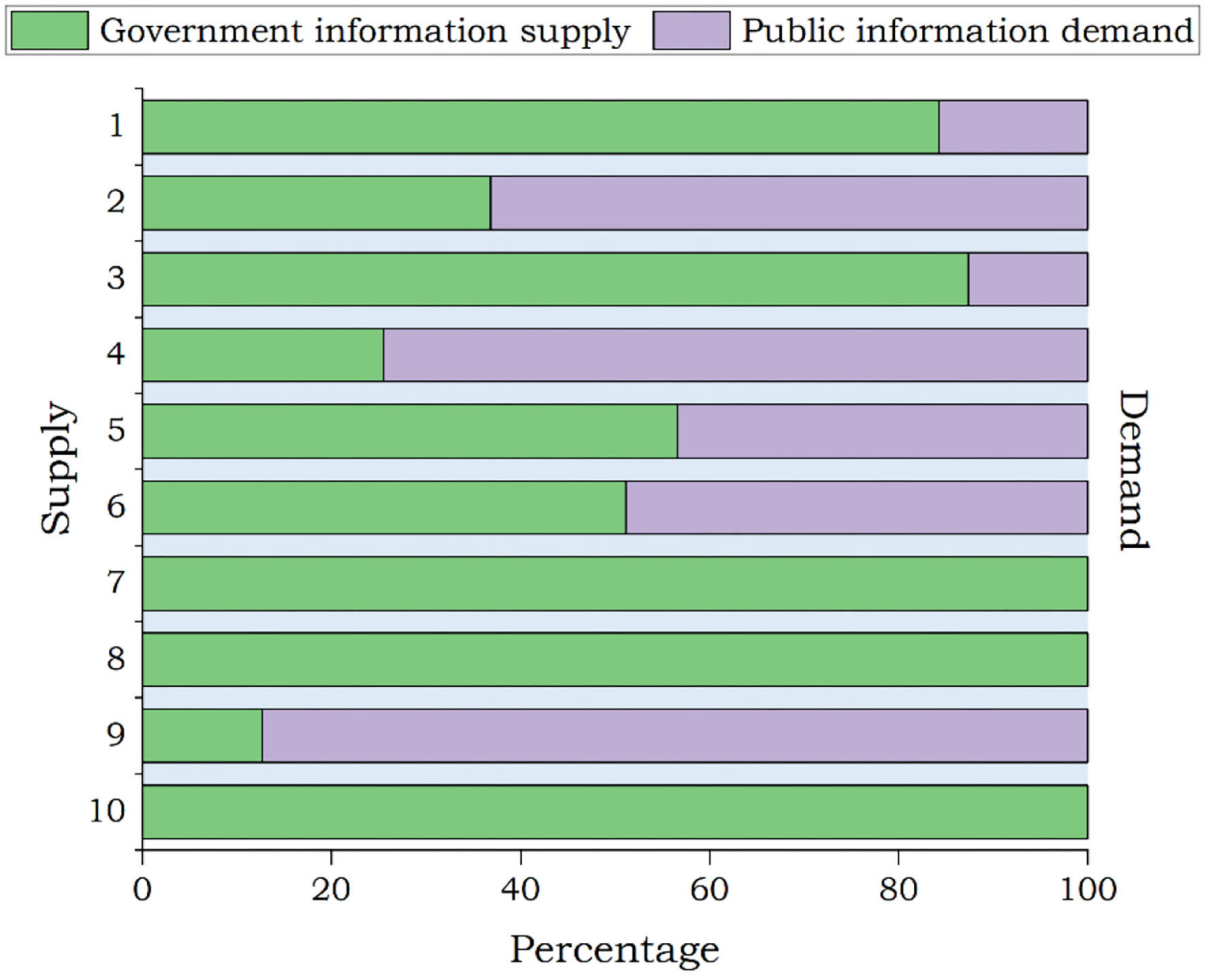
Outbreak period: government information supply and public information demand.

The differences between government information supply and public information demand during the outbreak period are presented in Figure 4. The theme with the most posts, i.e., “How beautiful you are,” did not attract the highest public attention, which is given in Figure 4, which showed a great gap between the government information supply and the public information demand. The theme with the highest public attention was “COVID-19 scientific knowledge,” and the corresponding number of government posts ranked second, of which the gap between supply and demand was small. By comparison, the public paid great attention to the “latest situation of the pandemic” and the “The pandemic policy,” while the supply of government information was limited. All the posts on these two themes released by the government had received high public attention. It is observed that the information supply and demand of these two themes, i.e., “National instructions” and “A healthy and happy New Year,” were balanced, and the matching degree of information supply and demand was high.

### Supply and Demand of Public Health Information During Stable Period

As the number of new cases of infection in China were gradually dropped below 10 (February 21, 2020 to March 17, 2020), the government made decisions to coordinate pandemic prevention and control, economic and social development and to resume work and production in an orderly manner. During the pandemic control period (from February 16, 2020 to March 05, 2020), the government has released 717 posts that all were related to COVID-19 (as shown in Table 6).

**Table 6:** The release frequency of government-run WeChat account and the public's reading preferences.

**Number**	**Keywords**	**Government**	**public**
1	How beautiful you are (medical staff)	220	7
2	Joint prevention and control conference	144	9
3	COVID-19 scientific knowledge	115	46
4	Community level	68	3
5	National instructions	63	8
6	The pandemic policy	37	17
7	Work resumption	25	0
8	Latest situation of the epidemic	19	19
9	WHO	14	0
10	Mobile cabin hospital	12	1

In terms of public information demand, there were 8 posts with more than 100,000 views, 262 posts with 10,000–100,000 views, and 270 posts with more than 10,000 views, accounting for 38% of the total number of posts. At this stage, the public had paid certain but less attention to the pandemic information compared with the outbreak period (Table 7).

**Table 7:** The control period of single posts read 100,000 posts.

**Public focus**	**Marked posts**	**Release date**	**Primary coverage**
Medical staff	It's settled by the nation! To increase the salary, and to decrease the requirement for professional title evaluation and employment of front-line medical staff!	02/23/2020	Ten measures to care for front-line medical staff were issued by the nation.
The pandemic policy	Notice on the issuance and interpretation of COVID-19 Diagnosis and Treatment Protocol (Sixth Edition)	02/19/2020	The phased diagnosis and treatment protocols of COVID-19
International cooperation	The China-World Health Organization Joint Investigation Report on COVID-19 was released	02/29/2020	Novel coronavirus was an animal-derived virus; the intermediate host has not been identified;
The pandemic policy	The Guidelines of the Pandemic Prevention and Control Measures for Enterprises and Public Institutions during resumption of Work and Production was issued	02/22/2020	The Notice on the Joint Prevention and Control Mechanism of Novel Coronavirus Pneumonia by the State Council
Medical staff	Outstanding collectives and individuals in COVID-19 prevention and control of the national health system were honored by 3 national departments (list attached)	03/05/2020	The National Health Commission, the Ministry of Human Resources and Social Security, and the State Administration of Traditional Chinese Medicine issued the Decision on Honoring the Outstanding Collective and Individuals in COVID-19 Prevention and Control of the National Health System

**Figure 5:**
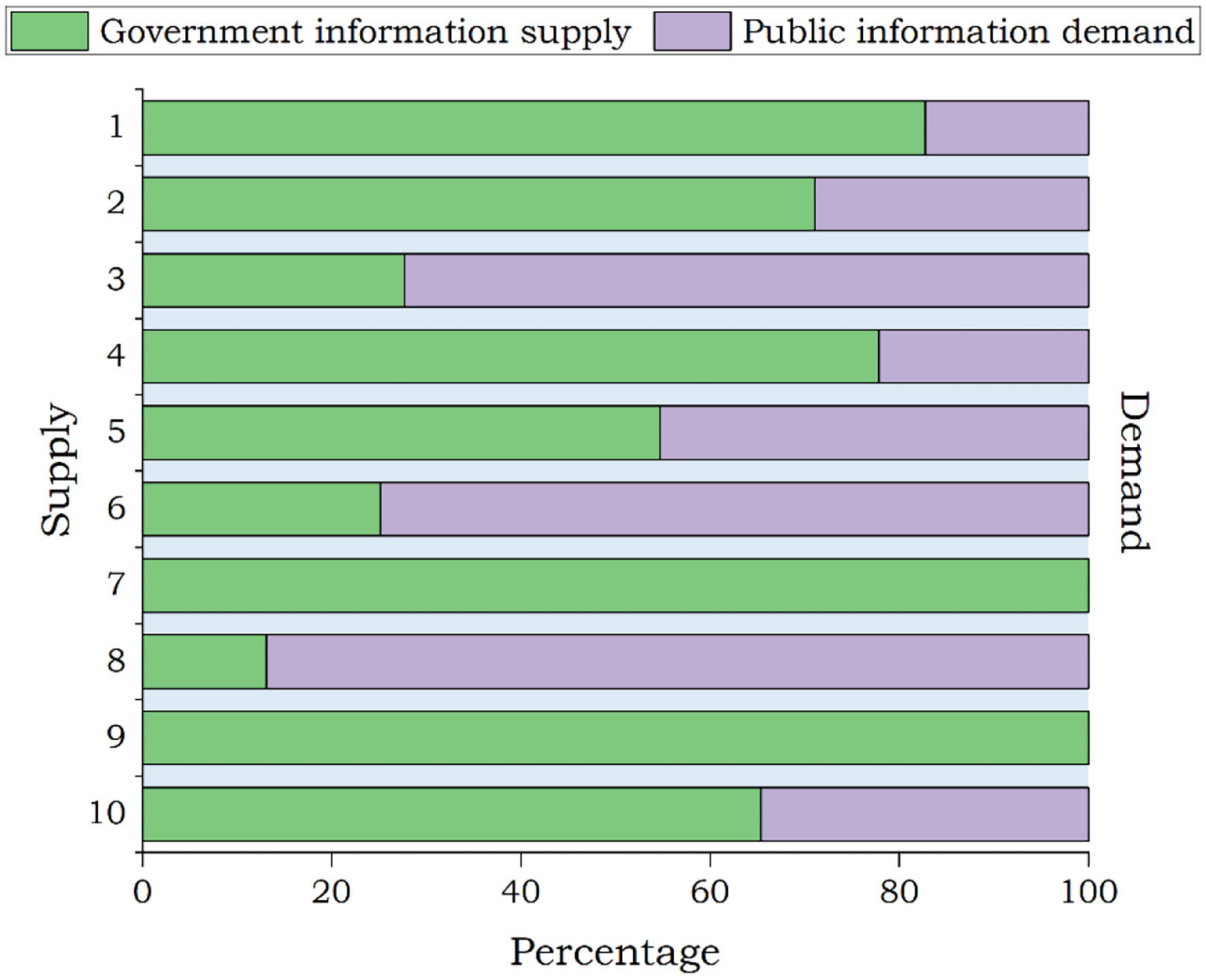
Pandemic control period: government information supply and public information demand.

In terms of theme, the top three themes with the most posts were “How beautiful you are,” “Joint prevention and control conference,” and “COVID-19 scientific knowledge.” During the pandemic control period, the public has paid most attention to these themes: COVID-19 scientific knowledge, the latest situation of the epidemic, and pandemic policies. It is observed that the themes that the public was concerned were basically the same as those in the previous stage. As is shown in Figure 5, the public pays more attention to “COVID-19 scientific knowledge,” “latest situation of the epidemic,” and “the pandemic policy.” At the same time, the supply of posts with themes of “how beautiful you are,” “Community level,” “World Health Organization (WHO),” and “Work resumption” has seriously exceeded the public demand.

### Supply and Demand of Public Health Information During the Recession Period

During the pandemic control stage (from January 20, 2020 to February 15, 2020), the number of posts by the government is 781, 6% of which involve COVID-19 (as shown in Table 8). There were only 3 posts that achieved over 100,000 views, 167 posts with 10,000–100,000 views, and 170 posts with more than 10,000 views, accounting for about 22% of all the posts. At this stage, the interest of the public to the pandemic information remained at a low level and continued to decrease compared with the pandemic control period.

**Table 8:** Frequency of government release of posts and public reading preferences during the recession period.

**Number**	**Keywords**	**Government**	**Public**
1	How beautiful you are (medical staff)	220	13
2	Joint prevention and control conference	144	15
3	COVID-19 scientific knowledge	115	31
4	National instructions	63	7
5	Community level	57	0
6	The pandemic policy	37	12
7	Latest situation of the epidemic	31	26
8	Work resumption	25	1
9	WHO	14	1
10	Mobile cabin hospital	14	4
11	Party organization	9	0
12	Oversea pandemic	9	3
13	Asymptomatic infections	5	4
14	Diagnosis and treatment protocols	1	0

**Figure 6:**
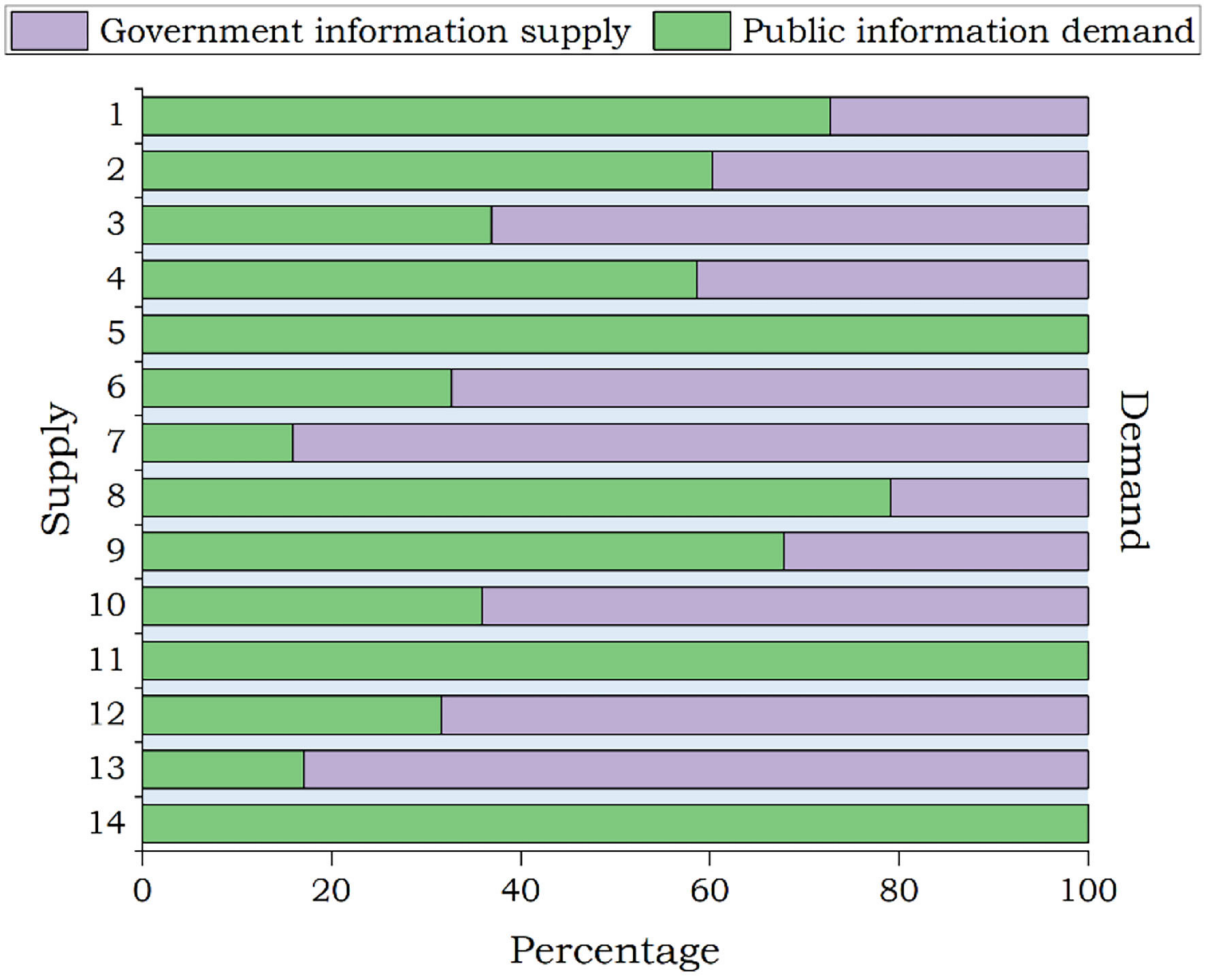
Government information supply and public information demand during the recession period.

Table 9 features posts attracting more public attention during the recession period. During the recession period of the pandemic, the top three themes with the most government posts were “How Beautiful You Are,” “Joint prevention and control conference,” and “COVID-19 scientific knowledge.” The themes that the public was most concerned about were “COVID-19 scientific knowledge” (such as mask-wearing guidelines, asymptomatic infections), “Latest situation of the epidemic,” and “Joint prevention and control conference.” As Figure 6 shows, during the period of the pandemic recession, the public has a high demand for information on various themes. Among them, themes with a high matching degree between government information supply and public information demand were “Joint prevention and control conference” and “The national instructions.”

**Table 9:** Posts with more than 100,000 views in the recession period.

**Public focus**	**Marked posts**	**Release date**	**Primary content**
The pandemic policy	Notice on the issuance and interpretation of public guidelines on mask wearing	3.18	The State Council issued guidelines on the selection and wearing of masks for different groups according to joint prevention and control mechanism for COVID-19
Scientific knowledge popularization	Official announcement: “Are the asymptomatic infection cases of COVID-19 infectious? Series Q & A are here!”	3.31	The official introduction of the COVID-19 asymptomatic infected person and characteristics
Scientific knowledge popularization	Twelve authoritative scientific questions and answers! [Scientific Knowledge Popularization of COVID-19]	3.11	The State Council press conference series of questions and answers on joint prevention and control mechanism of COVID-19
Scientific knowledge popularization	The post “personal protection and fighting against COVID-19” was released	3.25	Posts were used to display and Scientific Knowledge of COVID-19.
International cooperation	During a global pandemic, China held a special global conference	3.14	The WHO announced that the COVID-19 outbreak has constituted a global “pandemic.” China will report its experience to the world.

## Discussion and Conclusion

Based on the above analysis, the following conclusions and suggestions could be made. First, the National Health Commission maintained a very high intensity of information supply during the outbreak period, control period, and recession period, which reflected the great importance the Chinese government attached to the COVID-19 prevention and control work. In terms of the number of information supply, the average number of posts released by the government WeChat official account “Health China” was about 30 every day, far more than the average 2–3 posts during the non-pandemic period. The average number of daily posts could be 12 times that during the non-pandemic period. In terms of content, information supplied by the government presented characteristics with diversified themes and covered different aspects, such as “How beautiful you are” (medical staff), “COVID-19 scientific knowledge,” “Joint prevention and control conference,” “The pandemic policy,” “The national instructions,” “A healthy and happy new year,” “Latest situation of the epidemic,” “Party organization,” “Community level,” “Work resumption,” “Diagnosis and treatment protocols,” and “The world health organization.” Among which these, “How beautiful you are,” “COVID-19 scientific knowledge,” “Joint prevention and control conference,” and “The national Instructions” were four of the most prominent themes throughout all stages of the development of the pandemic. In addition, other themes also gained attention at different stages of the development of the pandemic. During the outbreak period, the “The pandemic policy” gained the most attention; and the “Community level anti-pandemic work” gained more attention during the pandemic control period and the pandemic recession period.

After the outbreak of COVID-19, the Chinese public had shown a strong demand for and paid attention to information related to the pandemic. In terms of stage characteristics, the public had the highest information demand during the outbreak period and gradually decreased during the pandemic control period and recession period. In terms of the content, the public reading preference presented a relatively stable characteristic. During all stages of the evolution of the pandemic, the public was concerned about the information of these 2 themes “Latest situation of the epidemic” and “COVID-19 scientific knowledge” most. During the outbreak period and control period, the public has paid more attention to the “The pandemic policy”; during the recession period, the public has paid more attention to the “Joint prevention and control conference.” Compared with other themes, the theme of “Community level” did not received high public attention at all stages of the development of the pandemic. In addition, the WHO also did not attract high public attention during the outbreak period and pandemic control period. In the evolving process of public health emergencies, there were certain gaps between government information supply and public information demand. “The national instructions” was the theme of the highest matching degree between supply and demand throughout all stages of the pandemic development. “The World Health Organization,” “Party organization,” “How Beautiful You Are,” and “Medical team” were themes of the lowest matching degree between information supply and demand. The matching degree between information supply and demand of these themes: “Joint prevention and control conference” was at a sub-low level. The reason for the low matching degree could be caused by the excessive number of posts by the government and the lack of public attention, resulting in the oversupply of information on a certain topic. It is observed that, unlike the classic agenda setting theory in the era of mass communication, the public's reading preference was independent to some extent and not affected by the number of posts by the government under a circumstance of wide and diverse information sources. Interestingly, in addition to the abovementioned themes with high frequency, a short supply of public's information demand has also occurred. For example, the public demand for information on the theme of “anxiety” during the outbreak period was much higher than what the government provided.

Based on the above findings, we propose three suggestions for the management of government-run official accounts during public health emergencies. Firstly, during public health emergencies, it should be of the first principle that the government release the latest trends in a timely and efficient manner to meet the urgent information needs about emergencies of the public. Secondly, the government should continue to provide scientific knowledge related to public health events to respond to the major concerns of the public. On the one hand, health-related knowledge is essential to help the public improve their awareness and strengthen their own pandemic prevention. On the other, mental health information is also necessary to help the public maintain both physical and mental health in special periods. Thirdly, in terms of public opinion guidance, too much information on themes of “how beautiful you are” and “anti-pandemic at the community level” could lead to a serious imbalance between supply and public information demand. Guiding the public opinion while avoiding public aversion is still a big challenge deserving future exploration.

## Data Availability Statement

The raw data supporting the conclusions of this article will be made available by the authors, without undue reservation.

## Author Contributions

ST and XW: conceptualization, methodology, software, data curation, and writing-original draft preparation. ZZ, YX, and JZ: writing-original draft preparation and funding. JC and FL: data curation, writing reviewing, and editing. All authors contributed to the article and approved the submitted version.

## Funding

This work was supported by the China Postdoctoral Science Foundation (2020T130102ZX), the Natural Science Foundation of Zhejiang Province (LQ21H190004), the Postdoctoral Science Foundation of Zhejiang Province (ZJ2020031), the National Natural Science Foundation of China (71873014), and the Fundamental Research Funds for the Central Universities, China (FRF-BR-20-04B and QNXM20210048).

## Conflict of Interest

The authors declare that the research was conducted in the absence of any commercial or financial relationships that could be construed as a potential conflict of interest.

## Publisher's Note

All claims expressed in this article are solely those of the authors and do not necessarily represent those of their affiliated organizations, or those of the publisher, the editors and the reviewers. Any product that may be evaluated in this article, or claim that may be made by its manufacturer, is not guaranteed or endorsed by the publisher.
